# Modeling knot features using branch scars from Mongolian oak (*Quercus mongolica*)

**DOI:** 10.7717/peerj.14755

**Published:** 2023-01-31

**Authors:** Xiu-jun Lu, Lei Wang, Hui-lin Gao, Hao Zhan, Xiao-lin Zhang

**Affiliations:** 1 Shenyang Agricultural University, Shenyang, China; 2 Chinese Academy of Forestry, Beijing, China

**Keywords:** X-ray, *Quercus mongolica*, Branch scar, Knot, Morphology models

## Abstract

Wood quality is an important indicator for modern sawmills. Internal wood characteristics can be derived from their correlations with external appearances. In this study, we developed linear regression models to predict knot size from surface features of Mongolian oak (*Quercus mongolica*) using data collected from 53 trees. For this, manual measurements and X-ray computed tomography scanning technology was respectively used to obtain internal and external features of 1,297 knots. Our results showed that Mongolian oak knots were generally concentrated in the middle part of oak stems, with fewer knots observed at the top and base. The parameters of knot and scar showed significant correlations (*P* < 0.01), where length and diameter of the corresponding external scar increase with increasing the length and diameter of a knot. The corresponding external scar can be used as an effective indicator to predict the internal value of oak logs. The accuracy of our constructed model is more than 95% when assessed against independent test samples. These models thus can be applied to improve the practical production of oak timber and reduce commercial loss caused by knots. These additional data can improve the estimation of the influence of knots on wood quality and provide a theoretical foundation for investigating the characteristics of hardwood knots.

## Introduction

Mongolian oak (*Quercus mongolica*) is a deciduous tree species from the genus Quercus commonly found in eastern Russia, northern China, and other northern Asian countries. It is one of the main building group tree species in northeast China and also a precious timber species, with important scientific value and development potential. Mongolian oak wood is used to make furniture, Mongolian oak acorns can be used as an energy plant and woody food, and Mongolian oak leaves are employed for the breeding of *Antherea pernyi* (Xu & Wang, 2002). The volume of Mongolian oak harvest in Russian has exceeded the national quota (Smirnov et al., 2013), which led to the species being placed on Appendix III of the Convention on International Trade in Endangered Species of Wild Fauna and Flora (CITES). Mongolian oak wood are commonly being used in ship construction, building, bridges, mechanical parts, *etc*. However, previous observations have shown a strong effect of winter apical bud death on Mongolian oak growth (Collet, Colin & Bernier, 1997). Usually, the loss of apical bud can increase side branch production, an incident that is detrimental to the cultivation of high-quality and large-diameter Mongolian oak wood (Han, 2019). This includes the formation of tree knots within a stem when side branches fall off the stem regardless of manual or natural pruning. Tree knots contain both sound and loose knot portions. Loose knots are especially detrimental to wood processing because they can easily fall off and form holes (Vestøl & Høibø, 2001). These features affect the value and utility of Mongolian oak wood (Chang & Gazo, 2009). In addition to tree diameter, knot characteristics, including the number, size, and distribution of knots on a stem, are important determinants of log quality (Mäkinen, 2002; Alcorn et al., 2008; Lowell et al., 2014). For example, lumbers is graded based on their surface appearance, it becomes important to understand the implications of this surface measurement for internal (Lowell et al., 2014). In most cases, even a single knot can significantly bring down the grade of an oak board (DIN Standard, 2011). In China, the knot diameter of grade I wood is less than 20% of the diameter at breast height (DBH) (China Standard, 2017).

Both destructive and non-destructive methods exist for collecting knot data inside logs. Destructive detection is suitable for tree with uniform knot distribution and is commonly used for conifer species, such as *Pinus sylvestris* (Björklund, 1997), *Pseudotsuga menziesii* (Newton et al., 2012), and *Pinus koraiensis* (Jia, Hong & Li, 2020). However, these methods damage the commercial value of measured wood. Preserving internal knots of wood is necessary to ensure the reliability of data. Recently, non-destructive techniques for rapidly generating high-resolution data have been developed, such as infrared imaging, optical scanning, magnetic resonance imaging (MRI), and computed tomography (CT) using X-rays. Internal wood defects can be obtained by scanning logs using non-damaging techniques. Benson-Cooper et al. (1982) first used CT scan to detect internal defects of logs. The recent and rapid development in computer technology has significantly enhanced the utility and accuracy of CT for detecting tree knots. For example, an algorithm can automatically detect knots and directly describe the size of knots on coniferous trees, such as *Abies alba* and *Picea abies* (Longuetaud et al., 2012). So far, CT has been used to describe knot geometry for *Picea abies* (Andreu & Rinnhofer, 2003), *Pinus sylvestris* (Moberg, 2006; Fredriksson, 2012), *Picea mariana*, *Pinus banksiana* (Duchateau et al., 2013), *Picea glauca* (Tong et al., 2013), and *Pseudotsuga menziesii* (Osborne & Maguire, 2016). The studies showed a strong correlation between CT data of internal knots and terrestrial light detection and ranging (t-LiDAR) data of external scars (Staengle et al., 2014). CT has been shown to be suitable for the assessment of wood quality (Staengle et al., 2014). A novel feature-based tracking approach can classify, locate, and reconstruct knots of *Fraxinus chinensis* and *Quercus rubra* L*.* (Bhandarkar et al., 2006). The development of secondary knots can also be predicted when CT technology is combined with 3D visualization software, which provides new insights into the understanding of branching dynamics of *Quercus petraea* (Colin et al., 2010).

In modern sawmills, a series of sawing optimizations have been used to improve wood value yield. CT technology could be used to support the decision about optimal rotational angle of logs to maximise volume and value yield (Staengle et al., 2015). Rinnhofer, Petutschnigg & Andreu (2003) showed that spruce could obtain a potential gain of 23.7% in lumber value *via* optimal rotation. The maximum volume and value of European beech logs can be increased by more than 24% *via* 3D reconstruction (Staengle et al., 2015). Applying the optimal combination of different rotations and parallel positioning in red pine can produce an additional 17,300 m^3^ of boards per year and thus increase the potential annual income by US$ 3.7 million per year (Lundahl & Grönlund, 2010). European beech, jack pine, white spruce, and Scots pine can produce an additional 24%, 23%, 15%, and 5% of lumber value, respectively (Staengle et al., 2015; Belley et al., 2019; Rummukainen, Makkonen & Uusitalo, 2019).

This study aims to create a method that utilizes external log features to estimate knot morphology. Specifically, this research develops models to describe knot size, which can be linked to the characteristics of scar seals. The obtained knot model could predict wood properties from external logs featuresof Mongolian oak. The objective of this study was to create model as a tool for Mongolian oak internal log features prediction of standing trees by applying the internal log features.

## Materials & Methods

### Plant materials

The experimental sites were set up on stands of natural secondary *Q. mongolica* forests in the Xiajiapu Forest Farm, Qingyuan Manchu Autonomous County, Liaoning Province, China (125°12′34″, 42°11′9″). The slope, altitude, mean annual temperature, and mean annual rainfall of the farm were approximately 30°, 280 m, 8.8 °C, and 926.3 mm, respectively. The natural vegetation of stand is dominated by *Q. mongolica* in the tree layer, *Fraxinus mandshurica*, *Fraxinus rhynchophylla* and *Phellodendron amurense* in the understory layer. Ten plots (20 ×20 m each) were randomly established from *Q. mongolica* stand. Plots were away at least 10 m from each other. We chose 4-6 trees in one plot. A total 53 Mongolian oak trees between 55 and 72 years old were selected at random (Table 1). Mongolian oak is a long lasting tree that reaches at 81 years old in tree farming systems. In these research, Mongolian oak trees belong to the immature timber stage (40–60 years old). These trees were cut down after DBH was measured. We divided these trees into 436 one-meter-length logs. Here, only stem portions with a diameter >5 cm were retained and marked at both ends from the base of the stem for later analyses. The logs were then transported to Shenyang Agricultural University in Shenyang City for CT scanning. The experimental sites were approved by Xiajiapu Forest Farm and Qingyuan Manchu Autonomous County Natural Resources Bureau.

### External data of log measurements

**Figure 1:**
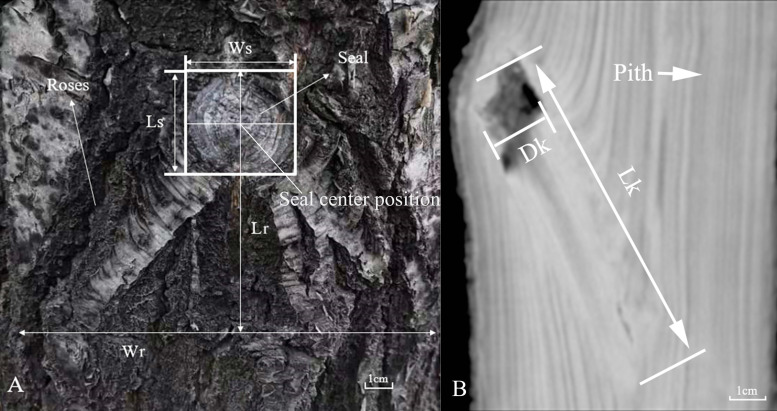
(A) Definition of scar (Ws, seal width; Ls, seal length; Wr, roses width; and Lr, roses length). (B) The occluded knot parameters on the CT image (Lk, knot length and Dk, knot diameter).

The insertion height of knot (IH); insertion diameter of knot (ID); seal width (Ws); seal length (Ls); roses width (Wr); and roses length (Lr) of 1,297 occluded knots (*i.e.,* knots that the sound and loose portions are partially occluded by the bark) observed on the bark surface of 607 logs were manually measured. These measurements were defined in Fig. 1A. Ws (horizontal direction) and Ls (vertical direction) are measured as straight lines between the edges of the seal. For each knot, we measured Ws and Ls three times, respectively, and retained their maximum values for analysis. The intersection of horizontal and vertical maxima is the seal center. Wr was measured as the straight line between the edges of two Roses. Lr was measured as the vertical distance between the upper edge of the scar seal and the lower edge of the scar seal. IH was measured as the height position of the knot along the trunk from the tree base. ID was measured as the trunk diameter at location of the knot occlusion (the seal center position). Data of each scar parameter were measured three times, and the average value was used. After log measurements were completed, all logs were scanned with CT immediately.

**Table 1:** Average attributes of Mongolian oak samples.

	DBH (cm)	Tree height (m)	Tree age (a)	Crown width (m)
Minimum	8.5	8.1	22	3.02
Maximum	19.2	16.3	45	10.5
Mean	13.2	11.2	32.6	5.6
Std. deviation	2.5	1.7	5.5	1.5
Std. error	0.05	0.03	0.10	0.03

### X-ray computed tomography for knots

Using 512 panoramic multimodal CT (NeuViz Epoch; Neusoft, Shenyang, China) devices, all logs were scanned. The CT equipment has a visual resolution of 0.17 mm, a one-time scan length of 110 cm, and a maximum scan weight of 100 kg logs. It logs at a rate of approximately two cm/s, and a complete scan takes 1–2 min. The thickness of the scanning plane was one mm; therefore, two consecutive CT images shared the same one mm thick slice of wood. Each 1-meter long log generated ∼1,000 consecutive CT images in this experiment. Using scan data, the CT equipment constructs a mosaicked 3D model in which the gray value of each voxel (3D pixel) represents the X-ray attenuation of a location in the log. The resulting CT image contains gray, black, and white tones that are represented in Hounsfield units. The accuracy of measurements depends largely on the color contrast of the image. Typically, a dark color indicates low wood density, and a white color indicates high wood density. Here, we only considered visible knots and adjusted the grey-level contrast to a fixed range (−1000 to +200 Hounsfield units) for all CT scans. The scanning voltage, current, window level, and window width were set to 120 kV, 100 mA, −450 HU, and 1,500 HU, respectively. We processed the CT images using Neusoft image diagnosis systems (DongSoft Inc., Shenyang, China), a professional software developed to handle CT images. The images of all knots were retrieved and measured directly from the NEUSOFT image diagnosis systems. This software has been demonstrated effective for CT image recognition and measurement. Knot shape was identified and extracted from a series of CT images using Multi-Plane Reconstruction (MPR) batch processing combined with basic morphology, threshold, and edge detection functions. Knot size was measured with a region of interest (ROI) tool (Fig. 1B) (Hou et al., 2020; Liu et al., 2018). Knot diameter was measured as the boundary between the sound and loose segments. Knot length was measured as the straight-line distance between the knot’s origin in the pith and the edge of the knot seal (Fig. 1B). The annual ring structure, part of the earlywood–latewood pattern, was also identified from CT images. Pith-to-bark profile was measured as the horizontal distance from pith to bark. Please see Table 2 for more details about these measurements. A total of 1,297 occluded knots were identified on the surfaces of sampled Mongolian oak trees. A total of 2,390 occluded knots were not visible on the surface. The mean values of knot IH, ID, Ws, Ls, Lr, and Wr were 5.56 m, 10.55 cm, 23.38 mm, 22.40 mm, 44.60 mm, and 56.51 mm, respectively (Table 3 and Table S1). On average, the number of knots per tree was 24. The minimum, maximum, and mean lengths of the sampled knots were 2.7, 41, and 11.36 mm, respectively. The minimum, maximum, and mean knot diameters were 2.1, 179.4, and 45.55 mm, respectively (Table 4 and Table S1).

**Table 2:** Definitions and abbreviations of variables used in this study.

Description			
Tree-level variables			
H	Total tree height (m)		
DBH	Diameter at breast height (cm)		
Branch-level variables			
IH	Insertion height of knot (m), the height position of the knot along the trunk from the tree base		
ID	Insertion diameter of knot (cm), the trunk diameter at location of the knot occlusion		
Ws	Seal width (mm)		
Ls	Seal length (mm)		
Wr	Roses width (mm)		
Lr	Roses length (mm)		
Knot-level variables			
Lk	Knot length (mm)		
Dk	Knot diameter (mm)		

**Table 3:** Mean attributes of scars.

	Minimum	Maximum	Mean	Std. deviation	Std. error
IH (m)	0.09	14.80	5.56	2.75	0.00
ID (cm)	1.10	19.30	10.55	2.50	0.00
Ws (mm)	1.18	92.74	23.38	12.15	0.01
Ls (mm)	0.70	82.67	22.40	12.20	0.01
Wr (mm)	2.16	174.78	56.51	23.08	0.02
Lr (mm)	1.01	154.41	44.60	22.38	0.02

**Table 4:** Mean attributes of knots.

	Minimum	Maximum	Mean	Std. deviation	Std. error
Lk (mm)	2.10	179.40	45.55	20.93	0.02
Dk (mm)	2.70	41.00	11.36	5.63	0.00

### Statistical analysis and model development

The SPSS 22.0 software was used in this study to analyze our data (IBM SPSS, Inc., Chicago, IL, USA). We used the least significant difference method (LSD) to perform multiple comparisons, with a significance level of 0.05. Meanwhile, stepwise linear regression analysis was applied to fit Eq. (1). The determination coefficient (*R*^2^) was calculated to evaluate the goodness of fit. We used an independent sample dataset to test the model, and the predictive ability of the model was evaluated using mean deviation (ME), mean absolute error (MAE), mean square error (MSE), root mean square error (RMSE), and prediction accuracy (*P*) (Li, Choi & Kim, 2001). At each modeling step, linear mixed effects model with individual tree as the random effect was used to fit the knot size distribution. All the parameters and combinations were used as the random effect and the model with the smallest AIC was selected as the best model.At each modeling step, a linear regression model was fitted. The general form of the multiple linear regression model is as below:

(1) \begin{eqnarray*}\mathrm{F(x)}={a}_{0}+{a}_{1}\mathrm{IH}+{a}_{2}\mathrm{ID}+{a}_{3}\mathrm{Ls}+{a}_{4}\mathrm{Ws}+{a}_{5}\mathrm{Lr}+{a}_{6}\mathrm{Wr},\end{eqnarray*}

where a0 is a constant term, and a1 to a6 are the regression coefficients of each independent variable.

## Results

### Patterns of knot distribution

**Figure 2:**
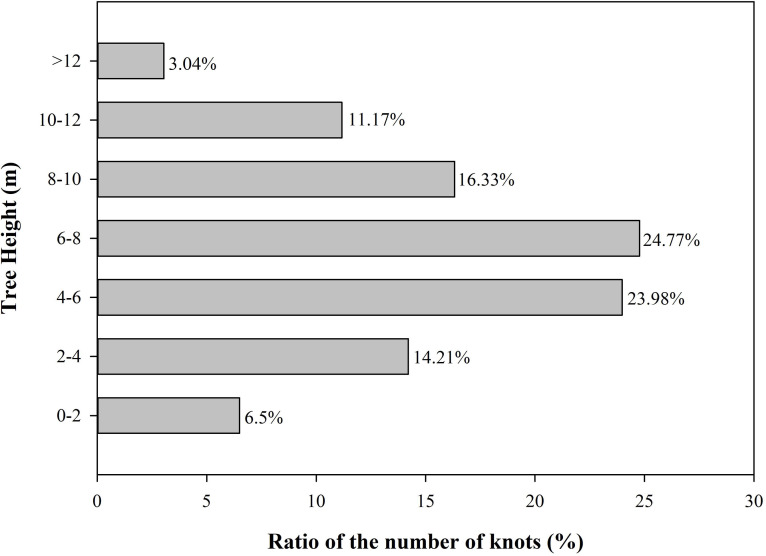
Proportion of knots along the tree heigh.

The number of oak knots increased first and then decreased with increasing tree stem height. The height range of 0–2 m contains a slightly lower number of knots compared with higher stem positions, accounting for 6.5% of the total observed knots. The number of knots increased at height 2–4 m and accounted for 14.21% of the total. The number of knots continued to increase at height 4–6 m and accounted for 23.89% of the total. The number of knots reached the maximum when stem height was 6–8 m and accounted for 24.77% of the total and then began to decrease with the continued increase of stem height. The number of knots accounted for 16.33% and 11.17% of the total when stem height was 8–10 and 10–12 m, respectively. The number of knots accounted for only 3.04% of the total when stem height was above 12 m. As shown in Fig. 2, approximately 48.75% of the identified knots were distributed within the height range of 4–8 m.

**Figure 3:**
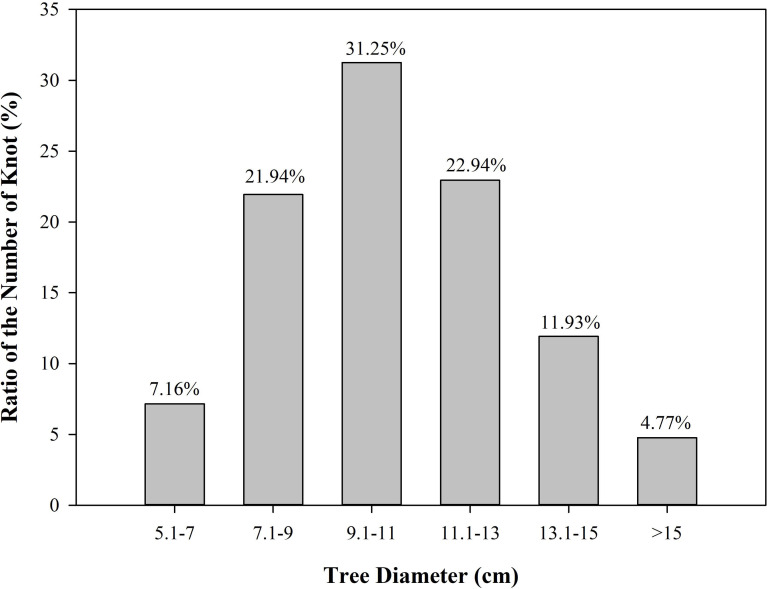
Proportion of knots along the tree diameter.

The number of Mongolian oak knots increased first and then decreased with decreasing stem diameter. The number of knots was slightly lower and accounted for only 4.77% of the total when stem diameter was larger than 15 cm. The number of knots began to increase and accounted for 11.93% of the total knots when stem diameter was between 13.1–15 cm. The number of knots continued to increase and accounted for 22.94% of the total when the diameter of the stem was in the range of 11.1–13 cm. The number of knots reached the maximum and accounted for 31.25% of the total when the diameter of the stem was in the range of 9.1–11 cm. Afterward, the number of knots began to decrease with a continued decrease of stem diameter (Fig. 3).

### Relation of internal and external features

Knot length was found to be positively correlated with seal length. Seal length increased with the increase of knot length when seal length was between 10 to 50 mm (Eq. 2: *y* = 20.7069 + 1.0778*x* + 0.0009*x*^2^  *R*^2^ = 0.6639). Knot diameter was also positively correlated with seal length. Seal length increased with the increase of knot diameter when seal length was between 10 to 50 mm (Eq. 3: *y* = 4.433 + 0.3053*x*  *R*^2^ = 0.6612). Knot length was positively correlated with seal width. Seal width increased with the increase of knot length when seal width was between 10 to 50 mm (Eq. 4: *y* = 21.6046 + 1.1044*x* + 0.0028*x*^2^  *R*^2^ = 0.5295). Knot diameter was positively correlated with seal width. Seal width increased with the increase of knot diameter when seal width was between 10 to 50 mm (Eq. 5: *y* = 4.097 + 0.348*x* − 0.0014*x*^2^  *R*^2^ = 0.5507). Knot length was positively correlated with roses length. Roses length increased with the increase of knot length when roses length was between 18 to 80 mm (Eq. 6: *y* = 21.0106*e*^0.0117x^  *R*^2^ = 0.7326). Knot diameter was positively correlated with roses length. Roses length increased with the increase of knot diameter when roses length was between 18 to 80 mm (Eq. 7: *y* = 6.3748*e*^0.012x^  *R*^2^ = 0.7114). Knot length was positively correlated with roses width. Roses width increased with the increase of knot length when roses width was between 20 to 100 mm (Eq. 8: *y* = 23.1534*e*^0.0113x^  *R*^2^ = 0.6775). Knot diameter was positively correlated with roses width. Roses width increased slowly with the increase of knot diameter when roses width was between 20 to 100 mm (Eq. 9: *y* = 5.3108*e*^0.0125x^  *R*^2^ = 0.709).

### Fitting and testing the Mongolian oak knot model

The measured scar parameters (IH, ID, Ws, Ls, Lr and Wr) were used as independent variables to conduct a correlation analysis with Lk and Dk (Table 5). Stepwise linear regression analysis was performed to determine the key factors that affect knot internal characteristics and construct the prediction model. A dataset of 317 knots from 13 trees was randomly selected as validation samples, and the remaining 980 knots from 40 trees were used to calibrate the prediction model (Table 6).

**Table 5:** Correlation coefficient between different variables.

	IH (m)	ID (cm)	Ws (mm)	Ls (mm)	Lr (mm)	Wr (mm)
Lk (mm)	−0.448^**^	0.481^**^	0.786^**^	0.865^**^	0.840^**^	0.768^**^
Dk (mm)	−0.399^**^	0.418^**^	0.428^**^	0.490^**^	0.498^**^	0.382^**^

**Notes.**

** The correlation is extremely significant when the confidence (double test) is 0.01.

**Table 6:** Data used in fitting and testing formulas.

	Formula fitting	Formula testing	Total
Number of trees	38	13	51
Number of knots	980	317	1,297

### Fitting the inner characteristic model of Mongolian oak knot

#### Knot length model

Our stepwise linear regression analysis showed that Lr, ID, Wr, Ls were closely related to Lk. These indices were then parameterized to construct the optimal model for describing Lk. The optimal model yielded an *R*^2^ of 0.64, with all input parameters significantly contributed to the model (*p* < 0.01) (Eq. (10)).

(10) \begin{eqnarray*}\mathrm{Lk}=3.833+0.434\mathrm{Lr}+1.029\mathrm{ID}+0.141\mathrm{Wr}+0.163\mathrm{Ls}.\end{eqnarray*}

Here, positive correlations were found between knot length and all four scar indices, which means a large external index value corresponds to a large knot length.

**Figure 4:**
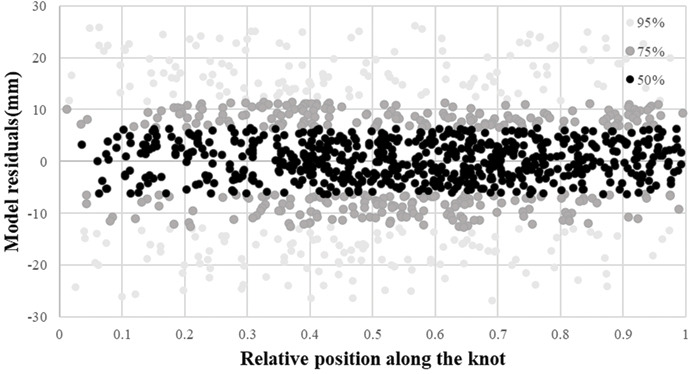
Residual distribution of the knot length model.

Knot length profiles were predicted and assessed against the evaluation dataset. The residual distribution of the knot length model showed that our knot length model was unbiased with values evenly distributed from 0.2 to 0.9 (Fig. 4). MAE and RMSE were 14.426 and 12.328, respectively (Table 7). The respective absolute values of 50%, 75%, and 95% of residuals were less than 6.5, 12.6, and 26.7 mm along with the pith-to-bark profiles for the knot length model (Eq. (10)) (Fig. 4). The quantile values are shown in Table S2.

### Knot diameter model

The knot diameter model yielded an *R* ^2^ of 0.59 throughout stepwise linear regression analysis. We optimized the model to determine the factors that best describe knot diameter (Eq. (11)). The fitting results showed that all used parameters were statistically significant (*p* < 0.01; Eq. (11)).

(11) \begin{eqnarray*}\mathrm{Dk}=1.377+0.101\mathrm{Lr}+0.064\mathrm{Wr}+0.061\mathrm{ID}+0.072\mathrm{Ls}-0.053\mathrm{IH}.\end{eqnarray*}

Similarly, positive correlations were observed between Dk and Lr, Wr, ID, and Ls, while knot diameter was negatively correlated with IH.

**Figure 5:**
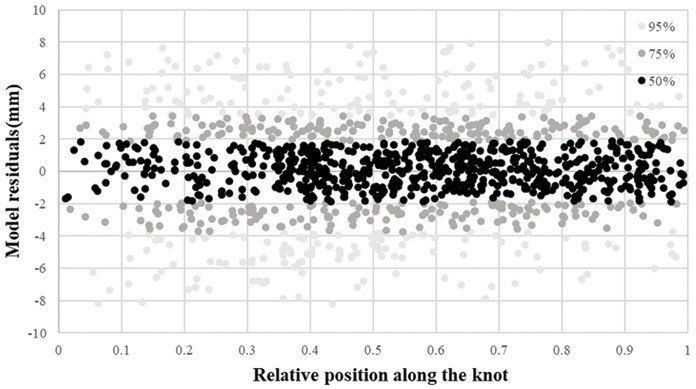
Residual distribution of the knot diameter model.

Knot diameter profiles were predicted and assessed against the evaluation dataset. The residual distribution of the knot diameter model showed that our knot diameter model was unbiased with values evenly distributed from 0.2 to 0.9 (Fig. 5). MAE and RMSE were 3.915 and 3.416, respectively (Table 7). The respective absolute values of 50%, 75%, and 95% of residuals were less than 1.9, 3.7, and 8.2 mm along with the pith-to-bark profiles for the knot diameter model (Fig. 5). Overall, a larger external index value indicates a larger knot diameter (Eq. (11)). The quantile values are shown in Table S2. Predicting the internal knot size using the external scar can be a potential and economical method. The development and utilization of external scars have gradually progressed, and the development of models for specific tree species can be a focus of future investigations.

**Table 7:** Test results of equations.

Model	ME	MAE	MSE	RMSE	*P* (%)
Knot length model	−0.901	10.787	253.393	15.918	96.90
Knot diameter model	−0.681	2.830	14.916	3.862	96.99

### Simulations

**Figure 6:**
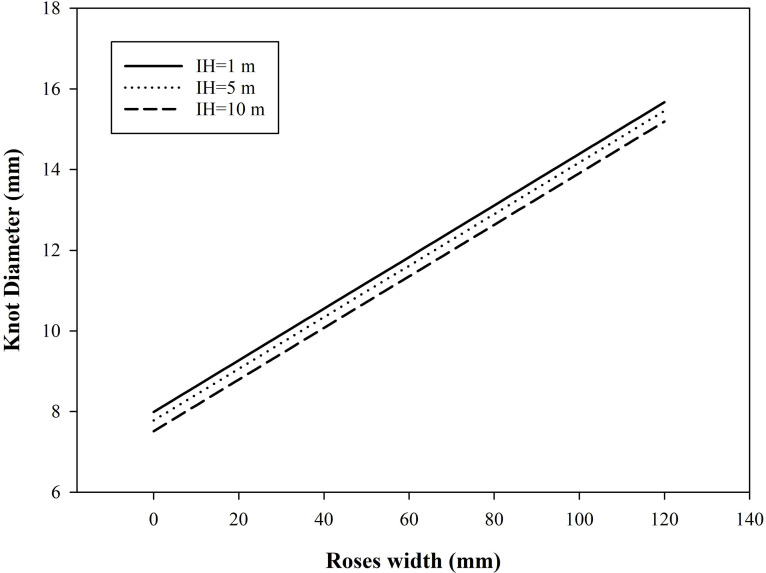
Knot diameter depicted by the model in Eq. (11).

To analyse the relationship of external log features on knot shape. Predictions of the models of knot length and knot diameter as a potential part of the growth simulation system are demonstrated in Fig. 6. The parameters were allowed to vary while all others were set to their mean values. Knot diameter increased with increasing roses width. The knot diameter was simulated at three different insertion height of knot (1 m, 5 m, 10 m). When the roses width was the same, the knot diameter decreased with the increase of insertion height of knot.

## Discussion

### Patterns of Mongolian oak knot distribution

Our results show that external features can be used to assess the wood quality of Mongolian oak in a detailed manner by leveraging the correlation between scar seal and knot.

CT technology was used to non-destructively determine the number of Mongolian oak knots. Scanning a 1-meter log took 2 min, which is much more time-saving compared to destructive methods. The manual validation measurements of scars parameters were time-consuming. Matching scars to the corresponding knots have been difficult because knots are usually closely located to each other. But manual measurement accuracy was usually higher than that of t-LiDAR technology which matched only 25% of knots to branch scars at knot heights lower than 30 mm (Staengle et al., 2014). In this study, all knots and scars were matched, possibly due to the high precision of manual measurements of external log features. CT-based automated knot detection methods have been developed for several softwood species (Grundberg, 1999; Oja, 2000; Longuetaud et al., 2012). Hardwood species also need automation in the wood processing industry.

The value of timber is affected by the size, quantity, and distribution of knots inside the log. Controlling where the knot appears in resulting boards is important when the log is sawed in a sawmill (Figs. 2 and 3). Knots should be avoided when they harm the structural integrity of the timber. Hence, the quality of the end product in the sawmill industry is highly dependent on the size, quantity, and distribution of knots (Zolotarev et al., 2020).

The knot size and distribution also have an important impact on wood product quality (Caceres, Uliana & Hernandez, 2018). In coniferous tree, the knot shows the most homogenous distribution of longitudinal strain (Buksnowitz et al., 2010). Usually hardwood trees do not have a homogenous distribution of knots along the stem (Staengle et al., 2014). The results of the two distribution trends can be mutually verified. The number of Mongolian oak knots first increased and then decreased with both stem height and diameter. Knots were found concentrated in the middle part of oak stems, with few found on the top and the base. This finding is similar to the distribution law of *Fraxinus mandshurica* (Zhuizhui & Yandong, 2020) and *Erythrophleum fordii* (Hao et al., 2017), but inconsistent with *Pinus sylvestris* (Chen, 2007) and Picea glauca (Tong et al., 2013), likely due to the difference in conifer species. Whorling and regular distribution characteristics demonstrated in branches of conifers are absent in hardwoods.

Knot size and the form of a knot (*i.e.,* sound or loose) determine the magnitude of the knot’s effects on wood quality. Loose knots are detrimental to oak wood quality. Hence, defining the knot structure may improve the utilization of wood (Vestøl & Høibø, 2000). However, this study is limited by its inability to define dead structure. Previous studies have identified sound and loose knots using their conical and cylindrical shapes, respectively (Samson, 1993; Trincado & Burkhart, 2008; Duchateau et al., 2013). Hence, we hypothesize that the point at which the maximum knot diameter is reached is at the boundary between sound and loose segments (Fig. 1B).

### Modeling knot internal characteristics using external log features

Previous studies have predicted internal characteristics of knots using branch diameter (Duchateau et al., 2013; Duchateau et al., 2015). However, branch stub becomes difficult to see after a branch loosens from the stem and new growth layers occlude the remaining parts of the branch. The features of scar seals are satisfactory estimators of stem diameter at the time of knot occlusion (Torkaman et al., 2018). Strong correlations between the shape of scar seals and internal defects have also been confirmed for other hardwood species (Thomas, 2009; Staengle et al., 2014). It is thus possible to predict wood properties from external logs. The shape of scar seals can be used to evaluate roundwood grades according to DIN Standard (2013). Hence, the production of high-quality timber determines the internal structure of logs. Scar seal quotient can predict the range of knot sizes in *Fagus sylvatica* (Staengle et al., 2014). This research successfully linked knot parameters to scar seal characteristics to improve the potential value of wood products. The results of this study showed that these external indices are significantly correlated with knot height and diameter (*P* < 0.01). The prediction model obtained from CT technology showed better performance than traditional measurement methods (Chen et al., 2015). In this study, independent samples were used to test the model, and the prediction accuracy was above 95%.

The application of this technology may be economically feasible in forest stands where high-quality and large-diameter Mongolian oak wood is commonly found, given the considerable differences in the price of Mongolian oak timber among different grades. Knots can be located in logs, and the sawing mode of logs can be optimized using Eqs. (10) and (11), respectively. Although the economic value of wood is affected by the market price (Steele et al., 1994), remarkable improvement is still possible (Chang & Gazo, 2009).

One of the limitations of this study is that the diameter at breast height was small (8.5–19.2 cm). In China, Mongolian oak occupies a significant area of secondary forests (Wang, Sang & Song, 1995). The rotation length for Mongolian oak is about 81 years. The samples used in this study belonged to young-to-middle age stands. Once a tree is exposed to competition with neighboring trees, it is then possible to promote DBH growth by removing its competitors. Forest thinning also have a harvesting function, providing intermediate income from small-sized products, before the final harvest (Thiffault et al., 2021). So our knot models could predict wood properties from external logs characteristics (young tree) and increase the gross value of lumber and volume yields of Mongolian oak.

## Conclusions

The results show that external log features can be used to assess the wood quality of Mongolian oak in more detail by employing the confirmed correlation between scar seal size and knot morphology. The scar seal size proved to be reliable when predicting the roundwood grading for Mongolian oak. These models could be used as an instrument to predict inner wood quality in greater detail by gathering data on the outer appearance regularly. this technique could provide for better silvicultural planning and more precise roundwood grading. In this research, the age of those trees did not reach harvesting. In future works, surveys should focus on testing with older trees to assess the applicability of the model to industry. Detailed external log features information can be gathered by manual or terrestrial laser scanning.

##  Supplemental Information

10.7717/peerj.14755/supp-1 Supplemental Information 1 Data for all knots

10.7717/peerj.14755/supp-2 Supplemental Information 2 The quantile values

## Additional Information and Declarations

### Data Availability

The following information was supplied regarding data availability: The raw measurements are available as a Supplemental File.
